# Neuromodulating roles of estrogen and phytoestrogens in cognitive therapeutics through epigenetic modifications during aging

**DOI:** 10.3389/fnagi.2022.945076

**Published:** 2022-08-03

**Authors:** Padmanabh Singh, Vijay Paramanik

**Affiliations:** Cellular and Molecular Neurobiology and Drug Targeting Laboratory, Department of Zoology, Indira Gandhi National Tribal University, Amarkantak, MP, India

**Keywords:** estrogens, phytoestrogens, epigenetic modifications, cognitive functions, brain

## Abstract

Estrogen (E2) plays important role in regulating hippocampal learning and memory. The decline of E2 after menopause affects learning and memory and increases the risk of neurodegenerative diseases like Alzheimer's disease (AD). Additionally, from the estrogen receptor (ER) mediated gene regulation; E2 also regulates gene expression at the transcriptional and posttranscriptional levels through epigenetic modifications. E2 recruits a number of proteins called co-regulators at the promoter region of genes. These co-regulators act as chromatin modifiers, alter DNA and histone modifications and regulate gene expression. Several studies show that E2 regulates learning and memory by altering chromatin at the promoters of memory-linked genes. Due to structural similarities with E2 and low side effects, phytoestrogens are now used as neuroprotective agents to recover learning and memory in animal models as well as human subjects during aging and different neurological disorders. Growing evidence suggests that apart from anti-oxidative and anti-inflammatory properties, phytoestrogens also act as epigenetic modifiers and regulate gene expression through epigenetic modifications. The epigenetic modifying properties of phytoestrogens are mostly studied in cancer cells but very little is known regarding the regulation of synaptic plasticity genes, learning and memory, and neurological disorders. In this article, we discuss the epigenetic modifying properties of E2 and the roles of phytoestrogens as epigenetic modifiers in the brain to recover and maintain cognitive functions.

## Introduction

Sex steroid hormone E2 exerts its action mainly through interaction with the intracellular estrogen receptors ERα and ERβ. In the classical mechanism of E2 action, the E2–ER complex interacts with estrogen-responsive elements (ERE) present at the promoter region of target genes and regulates their expressions. Apart from this, E2 also regulates gene expression through epigenetic regulation at transcriptional and posttranscriptional levels (Kovács et al., 2020) (Table 1). After binding at the ERE, the ligand-bound ERs recruit a number of proteins that act as co-regulators, i.e., co-activators and co-repressors (Thakur and Paramanik, 2009). Many of these co-regulators act as chromatin modifiers and thus regulate covalent modification of DNA and histone post translational modifications (PTM) (Marino et al., 2006). Several reports show that ERs interact with both the nuclear and mitochondrial proteins that are part of chromatin remodeling complex of the brain. The ligand binding domain (LBD) of ERα interacts with BAF60, a protein that is part of the SWI/SNF co-activator complex. BAF60 regulates chromatin remodeling through the hydrolysis of ATP (Ghosh and Thakur, 2009; Zhang et al., 2016). Similarly, LBD of ERβ interacts with cAMP response element binding protein (CREB) and pCREB, and helps in the recruitment of CREB binding protein (CBP). CBP is a histone acetyltransferase (HAT) and a part of p300/CBP co-activator complex (Thakur and Paramanik, 2009; Paramanik and Thakur, 2012).

**Table 1:** List of epigenetic mechanisms regulated by estrogens/phytoestrogens.

**Molecule**	**Epigenetic changes**	**References**
Estrogen (E2)	Increased histone PTMs (acetylation, methylation and phosphorylation) and demethylation on DNA in young and adult female's cortical slices.	Thakur et al. (1978); Kanungo and Thakur (1979a,b); Thakur and Kanungo (1981)
	Increased DNMT 3b expression, decreased HDAC2 expression, increased H3 and H4 acetylation and spatial memory in ovariectomized mice	Zhao et al. (2010)
	Regulated the expression of miRNA-233 in SH-SY5Y cells	Pan et al. (2021)
	Increased expression and stability of miRNA-9-5p and miRNA-9-3p in the brain and neuronal cell lines	Kim et al. (2021)
Estriol (E3)	Increased EZH2, SUZ12, CBP, DNMT1 and LSD1 expression in the hippocampus of adult offspring	Zhou et al. (2022)
Resveratrol	Increased DNMT1 and DNMT3a expression and attenuated learning and memory deficit in mice	Izquierdo et al. (2021)
Genistein	Increased HAT1 expression and H3K9Ac in prostate cancer cells	Phillip et al. (2012)
	Increased miRNA 132 expression and memory in ovariectomized mice	Scott et al. (2012)

E2 have known to play a key role in regulating hippocampal learning and memory, and the decline of E2 after menopause severely increases the risk of memory loss and neurodegenerative diseases in females (Viña and Lloret, 2010; Zárate et al., 2017). E2 replacement therapy (ERT) shows beneficial effects and improves learning and memory during aging. However, due to many side effects of ERT, alternative therapies are needed. Phytoestrogens (i.e., genistein and resveratrol) are widely used as therapeutic drug to recover learning and memory during aging, neurodegenerative diseases and various neurological disorders (Singh et al., 2021). Phytoestrogens are naturally occurring plant-derived secondary metabolites found in many food products such as soy and grapes. Due to their structural similarities with E2, phytoestrogens show a similar mechanism of action by interacting with ERs. Several reports show that phytoestrogens also regulate gene expression through chromatin modifications. However, most of these studies are found on the cancer cell lines and very few studies are reported on the brain (Zhang and Chen, 2011). Therefore, it is important to identify various mechanisms through which these phytoestrogens work. The present review discussed the chromatin-modifying action of E2 and the potential of phytoestrogens as chromatin modifiers with respect to learning and memory during aging.

## Chromatin modification and memory

Core histone proteins H2A, H2B, H3, and H4 along with 147 bp of DNAs and linker histone form the nucleosome. The N-terminal tails of histone protein are subjected to PTM on lysine (K), arginine (R), and serine (S). These PTMs include methylation, acetylation, sumoylation on lysine, phosphorylation on serine, methylation on arginine, etc. These PTM on histone influence chromatin structure and thereby regulate gene expression at the transcriptional level.

DNA methylation is catalyzed by DNA methyltransferases (DNMTs) that transfer a methyl (-CH3) to cytosine residues at the 5'C position of a CpG dinucleotide. DNMTs are categorized into de novo DNMTs (DNMT3a and DNMT3b) that methylate at previously unmethylated CpG and the maintenance DNMT (DNMT1) that methylates at the hemimethylated site on replicating DNA. DNA methylation both positively and negatively regulates gene expression at the transcriptional level that is dependent on the binding of the activator complex (Chahrour et al., 2008) or repressor complex (Moore et al., 2012; Lu et al., 2013) at the CpG dinucleotides of the promoter region of genes, respectively. Several studies show that DNA methylation is important for memory formation and consolidation. The expression of DNMT3a and DNMT3b was upregulated in the hippocampus after contextual fear conditioning and is involved in long-term memory formation (Levenson et al., 2006; Miller and Sweatt, 2007). Further, double knockout (KO) of DNMT1 and 3a showed impaired fear memory and spatial memory consolidation (Feng et al., 2010). DNMT1 deficiency decreased total DNA methylation level in the cerebral cortex and hippocampus, impairment of spatial and recognition memory consolidation during aging in wild-type and heterozygous DNMT1 KO mice (Elsner et al., 2013; Singh and Thakur, 2014).

Phosphoryaltion on histone catalyze by histone kinases that add phosphate group and histone phosphatases that remove phosphate group. The addition of phosphate group increases negative charge on histone and alters chromatin structure (Bannister and Kouzarides, 2011). Phosphorylation modification on histone positively regulates learning and memory. Chwang et al. (2006) reported that contextual fear conditioning increased histone H3S10 phosphorylation in the CA1 region of hippocampus and inhibition of MEK (MAP kinase/ERK kinase) impaired fear memory. Histone phosphorylation at H3S10 position is associated with positive gene regulation and hippocampal-depended long-term memory formation. Similarly, Gräff et al. (2012) reported higher cortical and hippocampal-dependent short-term and long-term memory formation depending on the upregulation of neuronal immediate early gene Zif268 due to higher H3S10 phosphorylation at the promoter region.

Methylation (mono, di, and tri-methylation) at N terminal tail of histone is an epigenetic mark that may positively or negatively regulate gene expression depending on the number of methyl groups (Berger, 2007; Kouzarides, 2007; Ng et al., 2009). Methylation on histone catalyze by histone methyltransferases that add methyl group and histone demethylases that remove methyl group. Several studies show that histone methylation involves in hippocampal-dependent long-term memory formation. Gupta et al. (2010) reported that the trimethylation at H3K4 position increased at the promoter of synaptic plasticity genes Zif268 and BDNF 1 h after exposure to the contextual fear conditioning. H3K9 dimethylation catalyzed by G9a/G9a-like protein (GLP) lysine dimethyltransferase negatively regulated gene expression and memory. Gupta-Agarwal et al. (2012) reported that G9a/GLP in inhibition in entorhinal cortex increased the expression of neuronal immediate early genes EGR1 and cFos and improved fear memory formation in rats. The enzyme EZH2 is the catalytic subunit of PRC2 repressive protein complex that add three methyl groups at H3K27 position and negatively regulate gene expression. EZH2 is highly expressed in the neuronal stem cells and helps in the proliferation of neuronal progenitor cells by inhibiting the expression of Pten during adult neurogenesis. EZH2 conditional knockout mouse shows reduced neuronal cell number and impairment of contextual fear memory and spatial memory (Zhang et al., 2014).

Acetylation on histone regulate by HATs and histone deacetylases (HDACs). HATs add acetyl group from Acetyl CoA on histone while HDACs remove the acetyl group from histone. Several reports showed that HDAC2 is upregulated in the hippocampus of old mice as well as by Aβ peptide in neurodegenerative disease mouse model (Gräff et al., 2012; Singh and Thakur, 2014). Higher HDAC2 level is associated with decreased dendritic spine numbers, reduced histone acetylation, expression of synaptic plasticity gene expression, and memory in mouse models (Guan et al., 2009). On the other hand, the expression of acetyltransferase CBP decreased in the hippocampus of old rats (Chung et al., 2002). Overexpression of CBP in the hippocampus increases the expression of BDNF and ameliorates spatial memory deficits in Alzheimer's disease (AD) mouse model (Caccamo et al., 2010). Alteration of these enzymes decreased histone acetylation levels at the promoter synaptic plasticity gene and downregulated their expression during aging (Singh and Thakur, 2018).

MicroRNAs (miRNAs) are small (21-24 nucleotides) non-coding single-stranded RNA molecule that regulates the expression of a target gene by pairing with the 3'-UTR region of its mRNA (Lai, 2002; Friedman et al., 2009). Research in human subjects and animal models showed that these miRNAs were found to be important for the brain. The miRNA-101 and miRNA-20b-5p play important role in the regulation of amyloid-beta (Aβ) synthesis in AD. Aβ peptide is generated as a proteolytic product of the amyloid precursor protein and is associated with the pathogenesis of AD. Vilardo et al. (2010) reported that the miRNA-101 has a binding site in the 3'-UTR region of the APP mRNA. Thereafter, inhibition of miRNA-101 increased the level of APP while lentiviral overexpression of miRNA-101 decreased the level of APP and Aβ aggregates in the hippocampal neurons culture. This suggested that miRNA-101 negatively regulates the expression of APP and Aβ load. Similarly, Wang et al. (2022) reported that the expression of miRNA-20b-5p altered in the cortex and hippocampus of AD patients. Further, *in vitro* result in human neuronal culture showed that the miRNA-20b-5p has a binding site at the 3'UTR region of APP mRNA.

## E2-mediated epigenetic modifications

E2-mediated epigenetic modifications in the brain were first explored by Thakur and Kanungo in the 1980s. In the *in vitro* experiments, they incubated slices of the cerebral hemisphere of female rats of different age groups in a buffer containing radio-labeled molecules and checked their incorporation on histone and DNA in the presence of E2. They observed that E2 stimulates acetylation, methylation, and phosphorylation on histone and demethylation on DNA in young and adults, but not in old females. Further, they also observed that E2-mediated higher histone acetylation is associated with higher transcription in the cerebral hemisphere slices as evident from higher incorporation of [^3^H] UMP in the RNA (Thakur et al., 1978; Kanungo and Thakur, 1979a,b; Thakur and Kanungo, 1981). Several studies show that E2 treatment is very effective when administered perimenopausal and beyond the critical window period, E2 fails to show its beneficial effects such as epigenetic modifications, gene expression, neuroprotection, and cognition in aged females (Daniel, 2013; Sinha et al., 2021). Therefore, Thakur and Kanungo may observe E2-mediated epigenetic changes in the cortical slices of young and adults, but not in old females.

DNA methylation plays an important role in the formation of long-term memory. Zhao et al. (2010) reported that intrahippocampal E2 treatment increased DNMT3a and 3b mRNA expression after 45 min and DNMT3b protein expression in the hippocampus and enhanced recognition memory in mice. Further, E2-mediated enhancement of recognition memory is inhibited by nonspecific DNMT inhibitor 5-Azacytidine (5-AZA) when co-administered with E2. As DNMT3b (de novo methyl transferase) methylates previously unmethylated sites, E2 may alter the methylation pattern at the promoter region of several genes and their expression during long-term memory formation. Several studies showed that E2 not only increased the expression of DNMT3b but also its activity (Vini et al., 2022). E2–ER complex recruits several chromatin modifiers such as histone deacetylase (HDAC) 1 and the repressor complex known as polycomb complex 2 (PRC2) at the ERE. The HDAC1 is a histone deacetylase that removes the acetyl group from the histone at the H3K27 position. This unacetylated H3K27 is recognized by EZH2 (active enzymatic component of PRC2) having methyl transferase activity and adds three methyl groups (H3K27me3) at that position. Later, DNMT3b recognizes H3K27me3 modification, recruited there and add methyl groups at the cytosine residue of nearby CpG island and repressed the gene expression (Kovács et al., 2020). This showed that epigenetic modifications work in a coordinated manner and regulate gene expression.

Several studies revealed that E2 regulates histone acetylation in two ways, i.e., recruiting the HATs at the promoter of synaptic plasticity gene and regulating the expression of HDAC2. Zhao et al. (2010) observed that the intrahippocampal administration of E2 downregulated the expression of HDAC2 at the protein level after 4 h of treatment in ovariectomized mice. This downregulation of HDAC2 was associated with higher acetylation of histone H3 and H4 and recognition memory in mice. Though the protein level of HDAC2 changed, the mRNA expression of HDAC2 was remaining unchanged. The post-transcriptional regulation of HDAC2 may be due to the microRNA (miRNA). One of the miRNA expression in the brain regulated by E2 is miRNA-233 (Pan et al., 2021). Leuenberger et al. (2016) reported that miRNA-233 has the binding site on HDAC2 mRNA. When over-expressed, the miRNA-233 downregulated the expression of HDAC2 at the post-transcriptional level in the pulmonary endothelial cells. This suggests that post-transcriptional regulation of HDAC2 may be important in E2-mediated chromatin modification and gene regulation. Sirtuins (SIRT 1-7) are NAD-dependent class III HDACs. They are localized to the cytoplasm, mitochondria, along with nucleus, and plays important role in metabolism, calorie restriction, and longevity as well as regulation of learning and memory (Herskovits and Guarente, 2013; Xu et al., 2018). Several reports showed that knockout or decreased SIRT1 expression reduced dendritic arborization and impaired hippocampal-dependent memory such as spatial, fear, and object location memory (Michán et al., 2010; Heyward et al., 2012). Griñan-Ferré et al. (2016) reported that environmental enrichment upregulated the expression of SIRT2 and 6 and improved recognition and spatial memory in aging mouse model. Similar to HDACs, the expression and function of SIRTs are also regulated by E2 in the brain. Guo et al. (2017) reported that the neuroprotective action of E2 due to cerebral ischemic damage in ovariectomized rats mediated through the SIRT1-MAPK pathway. d-Galactose administration in rodents induces inflammation and generates ROS, and memory impairment similar to neurodegenerative disease. Khan et al. (2019) showed that E2 improved learning and memory by ameliorating d-galactose-induced inflammation and oxidative stress. Further, they reported that E2 increased the expression of SIRT1 through ERα.

Previous study showed that E2 administration not only regulates the expression but also increased the stability and functions of miRNA-9-5p and miRNA-9-3p in the brain of aged ovariectomized female brain as well as in the neuronal cell lines (Kim et al., 2021). MicroRNAs regulated by E2 are miRNA-181, miRNA-9, miRNA-495, miRNA-7a, and miRNA-125a-5p in the hippocampus and are predicted to be regulating SIRT1, Nr3c1, GABRA1a, and BDNF (Rao et al., 2013). Castellano et al. (2009) reported that E2 up-regulates the expression of several miRNAs through ERα in breast cancer MCF-7 cells. Some of the miRNAs regulated by E2 are hsa-miR-542-3p, hsa-miR-424, hsa-miR-489, hsa-miR-98, hsa-miR-32, hsa-miR-19a, hsa-miR-20b, hsa-miR-92b, hsa-miR-101, etc. Oxidative stress and oxidative stress-induced apoptosis are common features observed in neurological disorders such as AD. Administration of H_2_O_2_ in cell culture generates oxidative stress and induces cell death. Pan et al. (2021) reported that E2 increased the expression of miRNA-223 and reduced apoptosis of H_2_O_2_-treated SH-SY5Y cells. Higher miRNA-223 downregulated the expression of FOXO3, a transcription factor that activates the transcription of many pro-apoptotic proteins.

Environmental endocrine disruptors such as phthalates and BPA mimic E2 action after binding with ERs. They showed adverse effects such as loss of pregnancy, changes in labor timing and birth weight of infants and alteration of miRNA in the human placenta and reproductive system (Strakovsky and Schantz, 2018; Cariati et al., 2020). Apart from these, recent studies showed that BPA also alters the epigenetic modification in the brain that leads to cognitive dysfunctions (Wolstenholme et al., 2011). Perinatal exposure (gestational day 7 to postnatal day 21) of BPA (50 μg/kg/day) altered the expression of synaptic proteins, epigenetic modifications, and anxiety-like behavior (Kumar and Thakur, 2017a). BPA altered mRNA expression of DNMT1 and 3a and decreased the level of 5meC in the cortex and hippocampus of 8-week postnatal pups. Further, they reported that the histone acetylation at the H3K9/K14 position increased both in the cortex and hippocampus; however, the expression of HDAC2 increased in the cortex whereas decreased in the hippocampus of postnatal pups (Kumar and Thakur, 2017b). Similarly, Malloy et al. (2019) reported that perinatal exposure (2 weeks before mating to postnatal day 21) of BPA (50 μg/kg/day) increased the expression of DNMT1 and TET2, an enzyme responsible for 5-hydroxymethylation of cytosine in the brain. On the other hand, prenatal exposure of BPA did not alter the level of 5meC in postnatal pups. Aiba et al. (2018) reported that prenatal (gestational day 6–17) exposure of BPA (200 μg/kg/day) did not significantly alter the level of 5meC in the hippocampus of 12-week postnatal pups. This may be due to different doses and duration of BPA exposure.

The steroid hormone estriol (E3) is a type of E2 that is synthesized in humans and other primates during pregnancy. E3 has weak E2 signaling and involves in the reproductive, immune, and CNS. A recent study by Zhou et al. (2022) showed that E3 also alters the expression of chromatin-modifying enzymes and reduced anxiety-like behavior in the offspring. They checked the expression of several enzymes responsible for DNA methylation, histone acetylation, and histone methylation. They reported that exposure of E3 increased the expression of histone methyltransferase Enhancer of Zeste homolog 2 (EZH2), histone demethylase Suppressor of Zeste 12 protein (SUZ12), and histone acetyltransferase CBP. Further, the expression of DNMT1 and histone lysine-specific demethylase (LSD1) increased moderately in the hippocampus of adult offspring. Interestingly, E3 treatment did not affect cognitive functions such as recognition and spatial memory, however, reduced anxiety in 6-month-old adult offspring.

## Phytoestrogen and memory

Phytoestrogens belong to a class of plant-derived compounds called polyphenols having structural similarities with the vertebrate steroid hormone E2. Phytoestrogens cross the blood-brain barrier and they exert their action by interacting with ERs and show both non-genomic and genomic actions. Due to their antioxidative, anti-inflammatory, and anti-apoptotic properties, phytoestrogens are widely used as neuroprotective drugs to recover as well as enhance memory in different animal models as human subjects. The phytoestrogen resveratrol shows its antioxidative properties by scavenging reactive oxygen species O_2_-, OH∙ and protects microglia-dependent β-amyloid toxicity in AD animal models by inhibiting NF κB signaling (Hano and Tungmunnithum, 2020). Resveratrol also improved glucose metabolism, hippocampal connectivity, and memory performance in old subjects and mild cognitive impaired patients (Witte et al., 2014; Cicero et al., 2019). Genistein and daidzein are two important phytoestrogens found in soy and soy-derived food. Daidzein supplementation increased adult hippocampal neurogenesis by increasing the number of neural progenitor cells as well as their dendritic arborization in middle-aged female mice (Yamada et al., 2016). Genistein is the most studied phytoestrogen with respect to the recovery of learning and memory in postmenopause female subjects as well as animal models. Intake of genistein in old females (65–85 years) enhanced the processing speed in Digit Symbol Substitution Test (Alwerdt et al., 2019). Wang et al. (2014) reported that genistein improved cognitive functions and spine density in late middle-aged and estropause female rats similar to E2. Studies in animal models and human subjects showed that genistein is also beneficial in the improvement of learning and memory in AD and other neurodegenerative conditions (Bagheri et al., 2011). Secoisolariciresinol, a phytoestrogen found in sesame, is shown to improve learning and memory during aging. Intake of secoisolariciresinol enhanced Mini-Mental State Examination-based cognitive functions in Dutch women after 20–30 years post-menopause (Franco et al., 2005). This shows that, in contrast to the E2 which is very effective when supplemented perimenopausal, phytoestrogen can be very effective in old females even after 20–30 years of E2 deprivation.

## Phytoestrogen and epigenetic modification

Apart from the anti-oxidative and anti-inflammatory properties, phytoestrogens also regulate gene expression in the brain. Research from the lab and other's works showed that phytoestrogens increased the expression of synaptic plasticity genes such as ERK1/2 and BDNF in the brain and thereby improves memory in different animal models similar to E2 (Fortress and Frick, 2014; Shojaei et al., 2017; Kurrey and Paramanik, 2021). E2 regulates the expression of synaptic plasticity genes through chromatin modifications, which intrigues us to check whether phytoestrogens have any role in regulating gene expression through similar pathways during learning and memory. Several reports show that phytoestrogens are also altering epigenetic modifications such as DNA methylation, histone PTMs, and miRNA regulation (Table 1). Phytoestrogens as epigenetic modifiers have been mostly studied in cancer cells. Genistein regulated DNMTs and DNA methylation of several genes related to stem cell proliferation and transcriptional regulation in prostate cancer (Bilir et al., 2017). Genistein also increased the HAT1 enzyme and acetylation at the H3K9 position in prostate cancer cells (Phillip et al., 2012). Further, treatment of resveratrol downregulated the expression and activities of DNMT1, DNMT3a and 3b in breast cancer cells as well as modulated HDAC and histone acetylation levels in different cancer cells (Kala et al., 2015; Fernandes et al., 2017). Studies also showed that these phytoestrogens are also effective in regulating epigenetic modifications in ER-negative cancer cells, which suggests that phytoestrogens may also act through different pathways other than ER pathway (Farhan et al., 2019). Few reports show that phytoestrogens alter gene expression and memory through epigenetic regulation. Genistein improved spatial memory and increased the expression of BDNF, IGF-1, and miRNA 132 in the hippocampus of ovariectomized mice (Habibi et al., 2017). The miRNA 132 enhanced learning and memory by downregulating p250GAP and modulating the actin polymerization in the dendritic spine (Scott et al., 2012). Izquierdo et al. (2021) reported that resveratrol attenuated learning and memory deficit in F1 progeny due to high-fat diet intake by mothers. Further, resveratrol increased the expression of DNMT1 and DNMT3a and DNA methylation in F1 progeny.

## Conclusion and future directions

**Figure 1:**
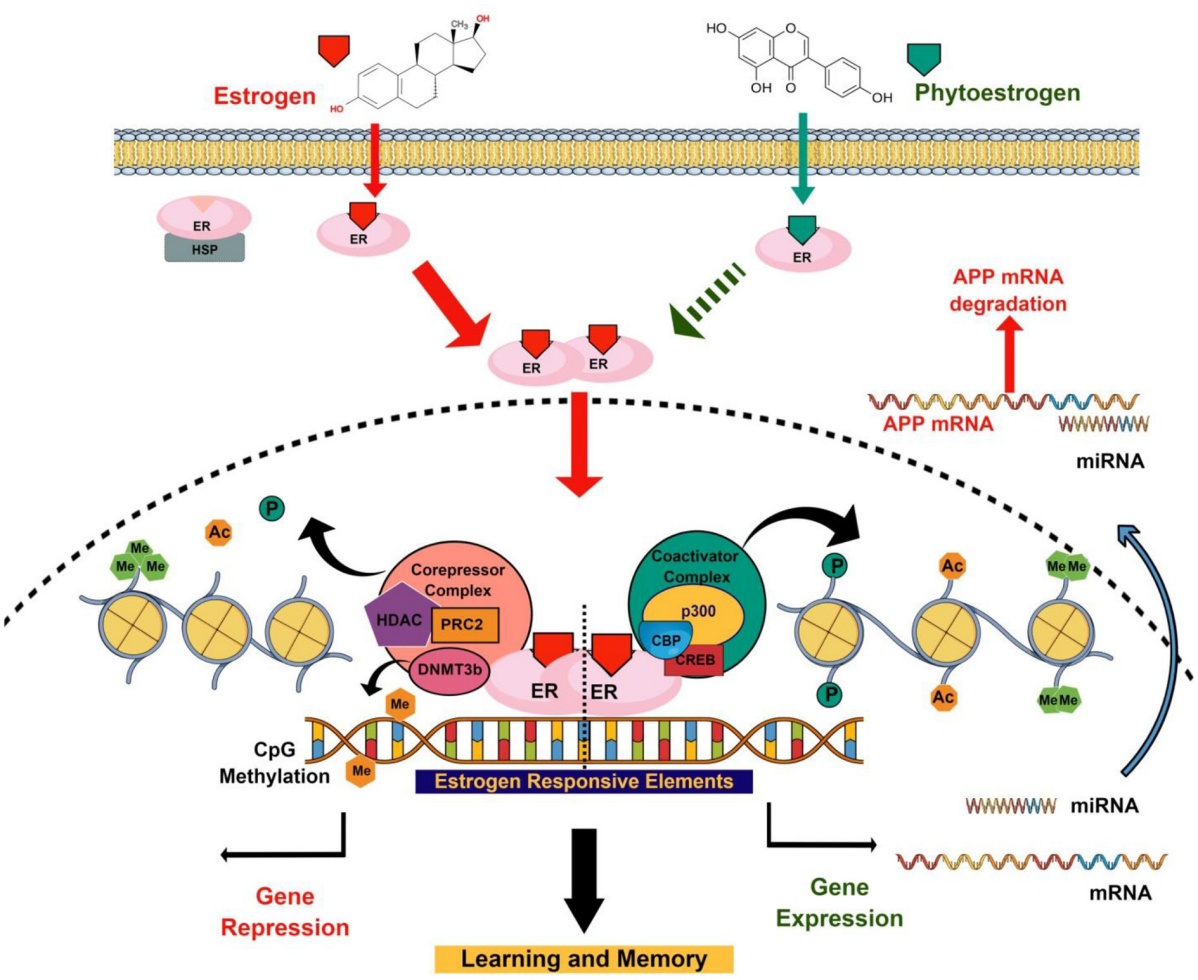
Schematic diagram representing neuromodulating roles of E2 and phytoestrogens through epigenetic modifications. E2–ER complex recruits co-regulators at the ERE of the genes involved in learning and memory. Co-regulators may be co-activator that induced gene expression and co-repressor that induced gene repression. These co-regulators regulate gene expression that involves learning and memory through epigenetic modifications (DNA methylation, histone PTMs, and miRNA). Similar to E2, the memory-enhancing properties of phytoestrogen may involve epigenetic modifications by recruiting coregulators at the promoter of synaptic plasticity genes.

Phytoestrogens show promising results for protecting the brain during aging as well as delaying the progression of neurodegenerative pathologies. Similar to E2, phytoestrogens also play important role in modulating epigenetic modifications, gene expression, and underlying functions (Figure 1). Phytoestrogens are used as an epigenetic modifier for a very long in cancer research, and their role as potential epigenetic modifiers with respect to brain and learning and memory during aging and neuropathological conditions are largely unexplored. Epigenetic modifications such as DNA methylation and histone PTMs are reversible in nature. Thus, modulating epigenetic modifications through phytoestrogens an alternative to ERT during aging to recover memory has significant therapeutic potential.

## Author contributions

PS and VP designed the study. PS prepared the manuscript. VP supervised the study and edited the manuscript. All authors contributed to the article and approved the submitted version.

## Funding

This work was supported by Department of Biotechnology (DBT-RA/2021/January/N/807) and Science and Engineering Research Board (SERB/LS-200/2013).

## Conflict of interest

The authors declare that the research was conducted in the absence of any commercial or financial relationships that could be construed as a potential conflict of interest.

## Publisher's note

All claims expressed in this article are solely those of the authors and do not necessarily represent those of their affiliated organizations, or those of the publisher, the editors and the reviewers. Any product that may be evaluated in this article, or claim that may be made by its manufacturer, is not guaranteed or endorsed by the publisher.
